# Team-based learning vs. lecture-based learning in nursing: A systematic review of randomized controlled trials

**DOI:** 10.3389/fpubh.2022.1044014

**Published:** 2023-01-04

**Authors:** Qin Zhang, Xiumei Tang, Yanjie Zhao, Zhoufeng Wang

**Affiliations:** ^1^Department of Postgraduate Students, West China School of Medicine, Sichuan University, Chengdu, Sichuan, China; ^2^Institute of Hospital Management, West China Hospital, Sichuan University, Chengdu, Sichuan, China; ^3^Department of Oncology and State Key Laboratory of Biotherapy, Cancer Center, West China Hospital, Sichuan University, Chengdu, Sichuan, China; ^4^Frontiers Science Center for Disease-Related Molecular Network, Institute of Respiratory Health, West China Hospital, Sichuan University, Chengdu, Sichuan, China

**Keywords:** nursing, team-based learning, lecture-based learning, education, effectiveness

## Abstract

**Introduction:**

Our study aims to identify, appraise, and summarize randomized controlled trials (RCT) on the effectiveness of team-based learning (TBL) versus lecture-based learning (LBL) in nursing students.

**Methods:**

We searched PubMed, Ovid, Embase, Cochrane, CBM, VIP, CNKI, and Wan Fang databases from inception to 22nd July 2022 to enroll RCTs that compared TBL versus LBL. The studies reporting the performance of nursing students receiving TBL pedagogy compared to those receiving traditional lecture-based learning (LBL) were to be analyzed. Scores of academic or nursing abilities were considered the primary outcome, and the results of nursing competencies, students' engagement with, behaviors, attitudes toward, experience, satisfaction, or perceptions of TBL were considered the secondary outcome. This systematic review was conducted following the guidelines of the Cochrane Reviewer's Handbook and the Preferred Reporting Items for Systematic Reviews and Meta-Analyses statement.

**Results:**

A total of 1,009 participants in 10 RCTs were enrolled in this study. Of the 10 RCTs, eight studies investigated undergraduate students, one involved vocational college students, and one enrolled secondary school students. The most reported outcomes were class engagement survey toward TBL (*n* = 8); students' ability (*n* = 5), academic knowledge or performance (*n* = 4); students' experience (*n* = 4), satisfaction or perceptions of TBL (*n* = 4).

**Conclusion:**

This review suggested that the TBL was an effective pedagogy in improving academic performance and general ability in nursing students. High-quality trials are needed, and standardized outcomes should be used.

## Introduction

Nurses are the most significant component of the healthcare workforce and take responsibility for multiple tasks, such as providing health promotion, counseling, and education; administering medications, clinical treatment, and other health interventions; taking part in critical decision-making; and research (1). The scope and complexity of nurses' work require deliberate educational preparation (2). Several national organizations have stated that traditional education methods, using lectures and relying on student memorization, centered on the unilateral delivery of knowledge, fail to adequately prepare nurses for current healthcare realities and call for new and innovative classroom models that are learner-centered and competency-based (3). Michaelsen initially invented team-based learning (TBL) in the 1980's to cope with the dilemma between faculty resource shortage and increased students (4). Usually, TBL contains a series of steps which include preparation, readiness assurance testing, feedback, and the application of knowledge through clinical problem-solving activities (5). One significant benefit of TBL is allowing large numbers of students to experience learning with a small number of expert facilitators. Students are motivated to complete the pre-reading assigned, resulting in less content being required to be covered during class. More in-class time is allocated to problem-solving and critical thinking, promoting greater understanding and retention of knowledge (6). TBL could help develop students' professional behaviors and improve learning outcomes through active learning and student engagement, ultimately enhancing students' ability in communication, teamwork, problem-solving, and critical thinking (6).

Recently, there have been a growing number of studies regarding the effectiveness of TBL in health professions {i.e., pharmacy (7), medicine (8), midwifery (9), and nursing education (10–14). A scoping review showed that TBL had been applied in nursing education over the last decade and reported outcomes involving students' knowledge/academic performance, student experience, satisfaction, or perceptions of TBL, student engagement, behaviors/attitudes toward TBL, and teamwork, team performance/collective efficacy (15). The significant gaps identified in this scoping review were the lack of RCTs, with only 3 out of 41 included studies being RCTs [dominant study designs were evaluation of TBL in isolation (*n* = 19)]. Moreover, systematic reviews have been conducted on the application of TBL in nursing education. However, their results were conflicted. Some researchers (12, 16–18) found TBL was not superior to a traditional lecture, while others found excellent results regarding TBL (10, 11, 13, 14, 19, 20). Among these studies, some were quasi-experimental designs (19, 21–25), some were one-group pre- and post-test designs (26–30), and some were cross-sectional investigations (31–35).

Randomized controlled trials (RCTs) have been considered the gold standard for effective research, but numerous reviews of studies of TBL report on the relative lack of evidence based on randomized studies. The most extensive examination to date of 118 studies of TBL in health professional education reported that 57% of studies compared TBL to another educational methodology while only one was an RCT (8). A systematic review of 17 studies enrolls one RCT, two prospective crossover studies, and ten descriptive, comparative studies (36). Notwithstanding, RCTs of TBL are desirable in establishing a high level of evidence for quantitative outcomes. To our knowledge, no systematic reviews evaluated the effectiveness of TBL based on high-quality evidence. We enrolled only RCTs to compare the efficacy of TBL to lecture-based learning (LBL).

## Materials and methods

### Search strategy

The review was reported according to the preferred reporting items for systematic reviews and meta-analyses (PRISMA) guidelines (37) and the guidelines described in the Cochrane Handbook (38). We searched PubMed, Ovid, Embase, Cochrane Library, CBM, VIP, CNKI, and WanFang databases from inception to 22nd July 2022. In addition to electronic databases, we also researched ClinicalTrials.gov and major international conferences. The reference lists of the retrieved papers were searched, and Google Scholar was used to search the gray literature. Search terms were related to nursing, education, and Team-Based Learning: the full search strategy is available in Appendix 1. No date limiters were set.

### Selection criteria

Inclusion criteria followed the PICOS principles: P, the participants were nursing students; I, the intervention was TBL pedagogy; C, the control method was LBL pedagogy; O, the outcomes included all the results reported in enrolled studies. S, the study design was RCT. There was no restriction on languages or publication years. The exclusion criteria were as follows: (i) editorials, letters, commentaries, opinion papers, case studies, case reports, unpublished theses, scoping reviews, systematic reviews, and meta-analyses and papers; (ii) participants were not in-college nursing students involving nurses or setting in a hospital. (iii) Studies where the implementation of TBL was not clearly described, incomplete or modified, and distance learning courses. Reference lists of potentially eligible studies and review articles were also searched to identify additional literature. Two authors independently screened records by titles and abstracts, and the other two read full texts of potentially eligible studies to determine eligibility. Any disagreements were resolved by consensus.

### Literature screening and data extraction

Two reviewers separately extracted the essential characteristics and the statistical data from articles that meet the above requirements. Conflicts were submitted to a third reviewer, and results were produced by comparison and discussion. If necessary, detailed statistics were asked directly from the corresponding author by E-mail. Each study's characteristics were extracted *via* a pre-defined checklist, including the first author, year of publication, number of students enrolled in each group, average years, and percentage of females. More detailed information was also collected, including country, recruitment period, courses (the content of TBL, teaching period, type of students, and staff numbers), outcomes examined, and significant findings.

### Quality assessment

Two authors independently rated the risk of bias in trials using the Cochrane Collaboration risk of bias tool (38). The study checked for random sequence generation, allocation concealment, blinding of participants and personnel, blinding of outcome assessors, incomplete outcome data, selective reporting, and other biases. The following domains were assessed for each study: selection bias, performance bias, detection bias, attrition bias, and reporting bias. The risk of bias table was completed using the Review Manager (RevMan 5.4) software. Discrepancies were resolved by consensus or discussion with the other authors. The level of discrimination was then classified as high, moderate, low, or very low.

### Outcomes

The outcomes were divided into four aspects: the primary product is academic scores or nursing abilities, which included examination scores, clinical performance scores, and in-class test scores. Secondary outcomes included: (i) nursing competencies: the competency can either be specific to a particular discipline or generic (such as community understanding or assessment of nursing abilities, clinical reasoning, critical thinking, problem-solving, clinical competence, communication competence, self-directed learning, and self-leadership abilities); (ii) student engagement, behaviors, or attitudes toward TBL [including the Classroom Engagement Survey (CES), learning attitude]; (iii) student experience, satisfaction or perceptions of TBL (group or peer evaluation, and students' perception of TBL).

## Results

### Search results

**Figure 1:**
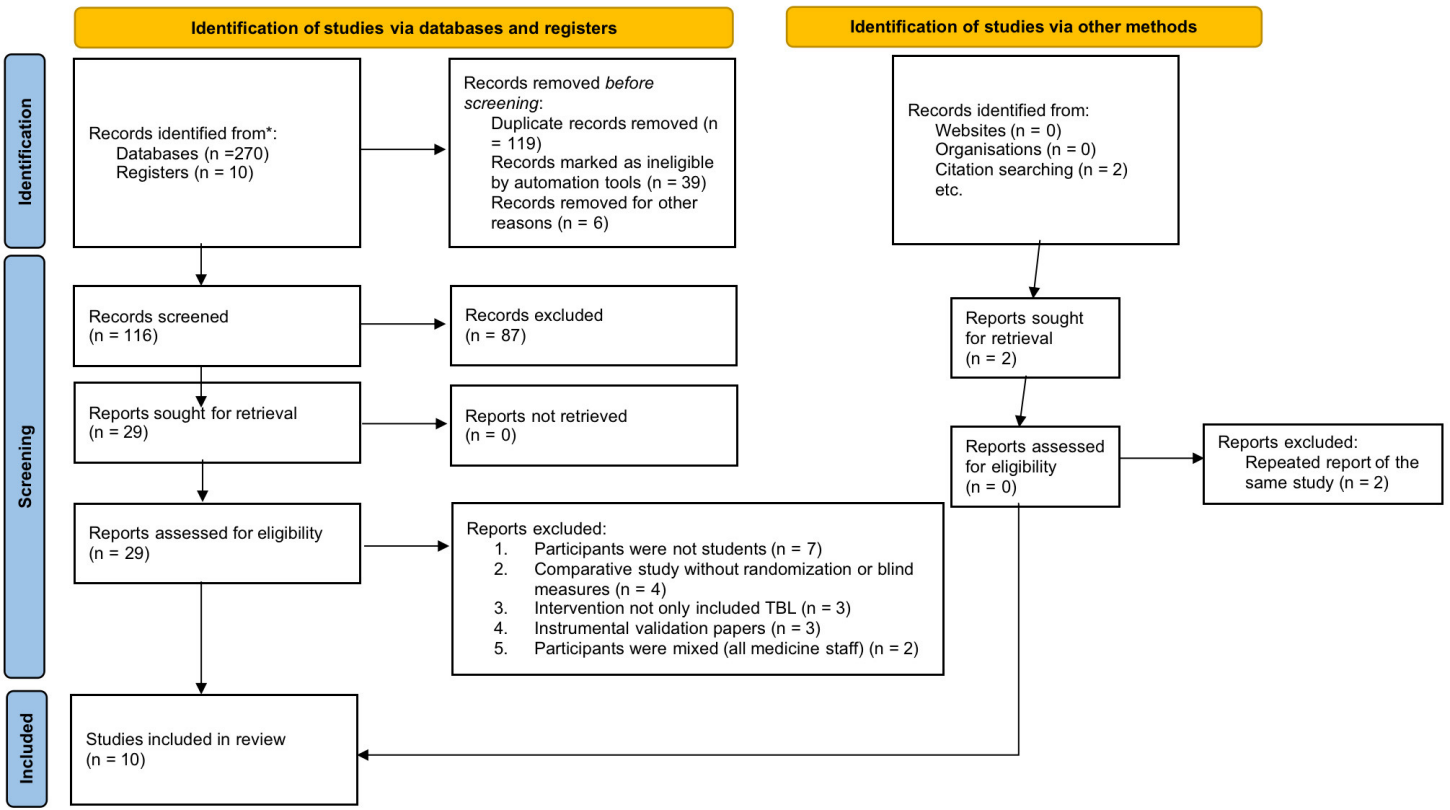
The search and selection process of included studies. The PRISMA 2020 statement: an updated guideline for reporting systematic reviews. (2021) 372:n71. doi: 10.1136/bmj.n71. For more information, visit: https://www.prisma-statement.org/.

The flowchart of the literature search and study selection is shown in Figure 1. The initial search yielded 290 results, from which 174 duplicates were removed, resulting in 116 unique records. Following the eligibility criteria, 29 relevant papers were identified based on title and abstract. This resulted in the final inclusion of 10 studies for analyses in this systematic review. No additional studies of relevance were found by searching the gray literature or hand-searching the reference lists of included articles.

### Baseline study characteristics

Ten studies published from 2011 to 2022 were identified for inclusion. Concerning the countries of the included studies, 4 of them were set in China (21, 39–41), 2 in Indonesia (10, 20), 2 in Korea (13, 14), one in Iran (42), and another one in Brazil (11). A total number of 1,009 participants were enrolled, and the sample size in each study ranged from a minimum of 25 (11) to a maximum of 183 (13) students. The total number of students in the TBL group was 523, and in LBL was 486. There was no difference in students' mean age or female percentage between the two groups (Table 1). Nineteen citations were excluded because participants were nurses with work experiences (*n* = 7); The study type was a comparative trial but not RCT (*n* = 4); the interventions were mixed with simulation teaching method (*n* = 3); instrument validation papers without data (*n* = 3); and the participants were interdisciplinary medicine students (*n* = 2).

**Table 1:** Baseline characteristics.

**Study (Year)**	**Patients (number)**		**Age‡**		**Female gender (%)**	
	**TBL group**	**Control group**	**TBL group**	**Control group**	**TBL group**	**Control group**
Yang et al. (21)	50	49	18.57 (0.51)	18.79 (0.98)	16 (32%)	44 (89.8%)
Ulfa et al. (10) A1	62	53	19.19 (0.54)	19.15 (0.50)	N/A	N/A
Ulfa et al. (20) A2	62	53	19.19 (0.54)	19.15 (0.50)	N/A	N/A
Sakamoto et al. (11)	14	11	21.80 (2.2)	20.70 (1.80)	24 (96%)	
Lee et al. (13)	95	88	23.57 (1.81)	23.48 (1.74)	89 (93.7%)	84 (94.5%)
Yang et al. (39)	55	51	22.62 (0.99)	22.57 (0.81)	43 (78.18%)	41 (80.39%)
Kim et al. (14)	32	31	22.25 (3.42)	22.39 (2.11)	4 (12.5%)	3 (9.7%)
Badiyepeymaie Jahromi et al. (42)	39	38	N/A	N/A	N/A	N/A
Xu et al. (40)	52	50	20.6 (0.9)	102 (100%)		
Han et al. (41)	62	62	N/A	N/A	51 (82.25%)	49 (79.03%)

‡ Data was presented as mean with standard error. N/A, not applicable.

For educational level, eight studies were of undergraduate students (10, 11, 13, 14, 20, 39, 41, 42), one was of vocational college students (40), and another one was of secondary school students (21). As for concerned topics, two studies featured in midwifery postpartum hemorrhage nursing (20, 21), two were about surgery nursing (11, 41), and the others were about pulmonary disease nursing (14), nursing core competency (13), emergency and intensive care nursing (40), community health care nursing (21), mental health and psychiatric disorders nursing (42), and geriatric nursing (39). TBL was implemented for varying lengths of time, ranging from a single session (11) up to courses that lasted for a whole semester (40). The number of academic staff was 2 in three studies (10, 11, 20, 40) and 3 in one study (41). In all the included studies, TBL was implemented according to the conceptual model's principles and main methodological phases. And all included studies used traditional lectures as the controlled measures except for one study, LBL lessons were combined with the innovative Web Quest method (42). In all the included studies, at least two different outcomes were assessed, of which at least one was measured quantitatively. Students' academic knowledge or clinical performance was most frequently mentioned and reported in 7 trials (11, 14, 20, 39–42); the students' experience, satisfaction, or perceptions of TBL were mentioned in 7 studies (11, 13, 20, 21, 39, 40, 42); generic competencies in terms of learning outcomes: instrumental competencies (i.e., problem-solving and critical thinking), communication and interpersonal skills (i.e., communication skills, self-leadership, interprofessional learning skills, and teamwork) and self-directed learning (or self-learning skills) were measured in five studies (10, 13, 14, 21, 39). More detailed information on the included studies is presented in Table 2.

**Table 2:** Study description.

**Study (Year)**	**Setting**	**Design**	**Recruit period**	**Courses**			**Tools/outcomes**	**Measure point**
				**Type**	**Period**	**Teachers**		
Yang et al. (21)	Taiwan	Comparative study	2020.9–2021.01	Junior college level; Nursing; Community health care nursing course;	6 weeks; 180 min per week	N/A	(1) TBL scale; (2) Learning attitude; (3) Nursing competence scale;	Pre-test, post-test
Ulfa et al. (10) A1	Indonesia	Cluster RCT	2019.09–2020.03	Bachelor level; Midwifery; Post-partum hemorrhage course;	3 weeks, 90 min per week	2	(1) PPH knowledge; (2) NSSS;	Pre-test, post-test, 2‵ 6‵ 12 weeks post-test
Ulfa et al. (20) A2	Indonesia	Cluster RCT	2019.09–2019.11	Bachelor level; Midwifery; Clinical reasoning and classroom engagement;	3 weeks, 90 min per week	N/A	(1) Clinical reasoning ability (*via* CREST); (2) CES;	Pre-test, post-test, 2 weeks post-test; 1‵ 2‵ 3 weeks post-test;
Sakamoto et al. (11)	Brazil	Cluster RCT	2017	Bachelor level; Nursing; Surgery safety knowledge;	1 lession, 120 min	2	(1) Learning investigation questionnaire; (2) Self and group evaluation;	Pre-test, 1 month post-test
Lee et al. (13)	South Korea	RCT	N/A	Bachelor level; Nursing; Adult health nursing course;	3 weeks, 120 min per week	N/A	(1) Nursing core competencies (clinical competence skills; problem-solving ability; communication competence measured by Global Interpersonal Communication Competence Scale; critical-thinking ability; self-leadership by Revised Self-Leadership Questionnaire)	Pre-test, post-test
Yang et al. (39)	China	Cluster RCT	N/A	Bachelor level; Nursing; Geriatric nursing courses;	1 semester	N/A	(1) Eysenck Personality Questionnaire; (2) SDL questionnaire; CTDI-CV; (3) Critical thinking; (4) Academical scores;	Pre-test, post-test
Kim et al. (14)	South Korea	RCT	N/A	Bachelor level; Nursing; Pulmonary disease course;	3 weeks, 120 min per week	N/A	(1) Problem-solving scale for college students; (2) 20-item multiple-choice questionnaire of participants' knowledge; (3) 13-item clinical performance checklist	Pre-test, post-test
Badiyepeymaie Jahromi et al. (42)	Iran	Comparative study	2013–2014	Bachelor level; Nursing; Mental health and psychiatric disorders courses;	N/A	N/A	(1) SDLRS; (2) Buford's self-regulation questionnaire;	Pre-test, post-test
Xu et al. (40)	China	Cluster RCT	2010.09–2011.02	Vocational level; Nursing; Emergency and intensive care nursing course;	18 weeks, 180 minutes per week;	2	(1) Academic scores; (2) Clinical performance; (3) Students' satisfaction;	Post-test
Han et al. (41)	China	Cluster RCT	2008	Bachelor level; Nursing; Urology surgery nursing courses;	1 lesson, 180 min.	3	(1) Academic scores; (2) Students' perception of TBL;	Post-test

RCT, Randomized controlled trial; TBL, Team-based lecture; PPH, Postpartum hemorrhage; NSSS, Nursing student satisfaction scale; CES, Classroom engagement survey; CREST, Clinical Reasoning Evaluation Simulation Tool; CCTDI, The California Critical Thinking Dispositions Inventory; SDL, Self-directed learning; SDLRS, Guglielmino's self-directed learning readiness scale; CREST, Clinical Reasoning Evaluation Simulation Tool. N/A, not applicable.

### Knowledge or clinical performance

The results of academic knowledge or clinical performance were measured in seven studies (TBL = 316; LBL = 296), and all the trials found that the exam scores were significantly higher following the implementation of TBL compared to the scores obtained from groups that received traditional lessons (Table 3). Kim et al. (14) (TBL = 32; LBL = 31) found that students in the TBL group had higher examination scores compared to those in the LBL group (TBL group 13.6 ± 3.2 vs. LBL group 12.0 ± 1.9, *p* < 0.05) at 1-week post-test. In Ulfa et al. (20) study (TBL = 62; LBL = 53), the knowledge of postpartum hemorrhage was measured at the immediate post-test, 2, 6, and 12 weeks *post-test*, and there were significantly higher scores in the TBL group (postpartum hemorrhage (PPH) knowledge at immediate, 2, 6, and 12 weeks *post-test*, all *p* < 0.001). Sakamoto et al. (11) (TBL = 14; LBL = 11) also found higher academic scores when measured at 1-month *post-test* (TBL group 7.2 ± 0.9 vs. LBL group 7.5 ± 0.9, *p* < 0.001). In the other four studies, the measurement time of academic scores was not mentioned, but the results significantly favored the TBL group. Other than examination scores, Xu et al. (40) (TBL = 52; LBL = 50) reported the performance of clinical skills (TBL group 92.09 ± 1.79 vs. LBL group 89.86 ± 1.88, *p* < 0.01), and the results were also in favor of TBL.

**Table 3:** The outcomes of included studies.

**Study (years)**	**Results**				**Conclusions**
	**Outcomes**	**TBL**	**Control**	***P*** **-values**	
Yang et al. (21)	(1) Learning attitude: Team efficacy	4.51 ± 0.54	4.28 ± 0.57	*p* = 0.039^*^	The results demonstrated that TBL improved participants' community understanding and enhanced their skills for assessing and fulfilling community needs. The experimental and control groups differed significantly in their TBL performance, learning attitude, and nursing competencies. The performance of those who engaged in TBL was higher than that of those who engaged in TBL on all community issues. TBL appears to be a more effective method than TL in terms of achieving nursing students' knowledge objectives.
	Collaborative learning	4.49 ± 0.57	4.19 ± 0.60	*p* = 0.012^*^	
	Learning attitude	4.56 ± 0.39	4.50 ± 0.46	*p* = 0.516	
	Individual self-efficacy	4.21 ± 0.37	4.21 ± 0.65	*p* = 0.994	
	(2) Nursing abilities: Community understanding	4.04 ± 0.40	3.04 ± 0.46	*p* < 0.001^*^	
	Community assessment	3.96 ± 0.57	3.33 ± 0.70	*p* < 0.001^*^	
	(3) TBL scale: Collaborative tendency	4.44 ± 0.51	4.23 ± 0.52	*p* < 0.05^*^	
	Communicative tendency	4.55 ± 0.48	4.35 ± 0.62	*p* = 0.077	
	Problem-solving tendency	4.49 ± 0.51	4.18 ± 0.63	*p* = 0.01^*^	
Ulfa et al. (10) A1	(1) PPH knowledge at immediate post-test;	85.20 (7.58)	72.49 (14.74)	*p* < 0.001^*^	The findings showed that TBL is an effective active learning strategy to improve knowledge of PPH of Indonesian midwifery students before clinical practice exposure. TBL also resulted in a higher learning satisfaction score in the intervention group.
	PPH knowledge at 2 weeks post-test;	83.59 (10.08)	71.73 (13.96)	*p* < 0.001^*^	
	PPH knowledge at 6 weeks post-test;	80.36 (9.07)	69.09 (17.16)	*p* < 0.001^*^	
	PPH knowledge at 12 weeks post-test;	85.95 (6.16)	77.02 (12.79)	*p* < 0.001^*^	
	(2) SNNN	34.19 ± 3.26	19.81 ± 1.94	*p* < 0.001^*^	
	(3) Willingness to be a midwife	57 (91.9%)	41 (77.4%)	*p* = 0.03^*^	
Ulfa et al. (20) A2	(1) Clinical reasoning scores after test	38.0 (7.36)	28.55 (5.89)	*p* < 0.001^*^	The mean clinical reasoning on postpartum hemorrhage scores were significantly higher in the TBL students than in the LBL students at post-test (*p* < 0.001; Cohen's d = 1.41) and 2 weeks post-test (*p* < 0.001; Cohen's d = 1.50). The CES showed a significantly higher in the intervention group than in the control group.
	(2) Clinical reasoning scores at 2 weeks	34.0 (7.32)	23.81 (6.16)	*p* < 0.001^*^	
	(3) CES at 1 week post-test	33.53 ± 2.83	22.34 ± 2.50	*p* < 0.001^*^	
	(4) CES at 2 weeks post-test	33.61 ± 2.96	21.68 ± 1.62	*p* < 0.001^*^	
	(5) CES at 3 weeks post-test	34.03 ± 2.98	20.94 ± 1.77	*p* < 0.001^*^	
Sakamoto et al. (11)	(1) Group evaluation at pre-test	29.4 ± 6.4	19.9 ± 4.1	*p* < 0.02^*^	Students' apprehension of knowledge in the TBL group was significantly higher compared to the control group (*p* < 0.002) by considering the pre-test results. After 30 days, there was no significant difference between groups. The experience with the methodology was considered positive among students.
	(1) Group evaluation at 1 month post-test	30.6 ± 4	27.6 ± 5.9	Not significant	
	(2) Peer evaluation (Team evaluation, Self-evaluation)	No total score			
	(3) TBL questionnaire	No total score			
	(3) Academical scores at 1 month post-test	7.2 ± 0.9	7.5 ± 0.9	*p* < 0.001^*^	
Lee et al. (13)	(1) Clinical competence skills	75.28 ± 9.26	72.18 ± 7.51	*p* = 0.014^*^	The TBL group achieved significantly higher scores for clinical competence skills, communication competence, critical thinking ability, and self-leadership post-test than pre-test, whereas the LBL group achieved significantly higher scores for clinical competence skills and critical thinking ability at post-test than pre-test. After the intervention, the experimental group had significantly better clinical competence skills, communication competence, and self-leadership than the control group.
	(2) Self-leadership	132.01 ± 17.1	126.73 ± 14.36	*p* = 0.025^*^	
	(3) Problem-solving ability	74.76 ± 20.84	72.53 ± 16.89	*p* = 0.431	
	(4) Communication competence	60.62 ± 7.38	57.86 ± 6.24	*p* = 0.007^*^	
	(5) Critical thinking ability	101.6 ± 12.28	99.03 ± 10.18	*p* = 0.127	
	(6) Students' preference	2.10%	3.40%	Not significant	
Yang et al. (39)	(1) Self-directed learning ability (overall scores)	74.19 ± 7.92	69.76 ± 8.40	*p* = 0.006^*^	The application of TBL in the teaching of geriatric nursing courses for undergraduate nursing can improve students' autonomous learning ability and critical thinking ability.
	(2) Critical-thinking ability	301.18 ± 19.02	289.49 ± 28.53	*p* = 0.014^*^	
	(3) Academical scores	80.61 ± 4.88	78.47 ± 6.52	*p* < 0.05^*^	
Kim et al. (14)	(1) Problem solving ability at 1 week post-test	164.7 ± 8.4	145.2 ± 5.6	*p* < 0.001^*^	This study found that TBL improved problem-solving ability, knowledge, and clinical performance in third-year Korean nursing students. Active team discussions and feedback strategies used in TBL were effective in obtaining positive learning outcomes.
	(2) Knowledge at 1 week post-test	13.6 ± 3.2	12.0 ± 1.9	*p* < 0.05^*^	
	(3) Clinical performance at 1 week post-test	22.3 ± 2.6	16.3 ± 1.0	*p* < 0.001^*^	
Badiyepeymaie Jahromi et al. (42)	(1) Total self-directed learning (rank rate)	43.24	39.3	*p* < 0.01^*^	Participants' self- directed (self-management) and self-regulated learning differed between the two groups (*p* = 0.04 and *p* = 0.01, respectively). However, the scores related to learning (students' final scores) were higher in the WebQuest approach than in team-based learning
	Self-control (rank rate)	37.33	35.72	*p* = 0.73	
	Self-engagement (rank rate)	34.57	38.33	*p* = 0.76	
	Self-management (rank rate)	31.11	40.75	*p* = 0.04^*^	
	(2) Final examination scores	59.08 ± 6.43	67.08 ± 6.43	*p* = 0.02^*^	
Xu et al. (40)	(1) Academic knowledge scores	84.83 ± 5.62	81.70 ± 8.21	*p* = 0.028^*^	Students in TBL class were better than those in LBL class on practical skills assessment, theory test scores and analysis quiz. The feedback of teaching content, teacher factors, examination and evaluation, and the overall satisfaction in study group were better than those in LBL group.
	(2) Clinical performance	92.09 ± 1.79	89.86 ± 1.88	*p* < 0.01^*^	
	(3) Students' satisfaction	108.44 ± 9.97	103.72 ± 6.68	*p* < 0.01^*^	
Han et al. (41)	(1) Academic knowledge scores	84.7 ± 2.6	78.9 ± 3.2	*p* < 0.01^*^	The application of the TBL teaching model improved students' academic knowledge scores, and most of the students were in favor of TBL using in class.
	(2) Students' perception of TBL (Percentage)	82.3–93.5%			

* : p < 0.05.

### Competencies

The effect of TBL on competencies was reported in six studies (TBL = 333; LBL = 310). Communication competencies were mentioned in 2 studies (*n* = 33; TBL = 177; LBL = 168) (13, 21). In Yang et al. (21) study (TBL = 50; LBL = 49), the results were similar between the two groups (*p* = 0.077) while Lee et al.' study (TBL = 95; LBL = 88) favored TBL (TBL group 60.62 ± 7.38 vs. LBL group 57.86 ± 6.24, *p* ≤ 0.007).

The problem-solving ability scores were reported in 3 studies (TBL = 177; LBL = 168) (13, 14, 21). In Yang et al. (21) study, the results were in favor of TBL (TBL group 4.49 ± 0.51 vs. LBL group 4.18 ± 0.63, *p* = 0.01), the results were similar to Kim et al. (14) (TBL = 32; LBL = 31), which reported a higher score of problem-solving ability at 1-week *post-test* (TBL group 164.7 ± 8.4 vs. LBL group 145.2 ± 5.6, *p* < 0.001). While Lee et al.' study found similar problem-solving ability scores between TBL and LBL groups (*p* = 0.431).

The critical-thinking ability scores were reported in 2 studies (TBL = 145; LBL = 137) (13, 39), Yang et al. (39) (TBL = 55; LBL = 51) showed significantly higher critical-thinking scores (TBL group 301.18 ± 19.02 vs. LBL group 289.49 ± 28.53, *p* = 0.014) in the TBL group. However, the results in Lee et al.' study were similar between TBL and LBL groups (TBL group 101.6 ± 12.28 vs. LBL group 99.03 ± 10.18, *p* = 0.127).

The self-directed learning scores were reported in 2 studies (TBL = 55; LBL = 51) (39, 42), Yang et al. (39) showed significantly higher self-directed learning ability scores (TBL group 74.19 ± 7.92 vs. LBL group 69.76 ± 8.40, *p* = 0.006). Badiyepeymaie Jahromi et al. (42) (TBL = 39; LBL = 38) also found similar results (TBL group 43.24 vs. LBL group 39.3, *p* < 0.01).

Moreover, Yang et al. (21) divided nursing competencies into community understanding, community assessment, collaborative tendency, and problem-solving tendency, which all benefit the TBL group (all *p* < 0.05). Ulfa et al. (10) reported results measured at multiple time points and found TBL improved students' clinical reasoning scores right after the test as well as at 2 weeks *post-test* (immediately after the test: TBL group 38.0 ± 7.36 vs. LBL group 28.55 ± 5.89, *p* < 0.001; 2 weeks after the test: TBL group 34.0 ± 7.32 vs. LBL group 23.81 ± 6.16, *p* < 0.001). Lee et al.' study reported five subscales of clinical competence skills, including scores of self-leaderships, problem-solving ability, communication competence, and critical thinking ability. Among them, positive responses favoring the TBL group were evidenced in three outcomes (clinical competence skills, *p* = 0.014; self-leadership, *p* = 0.025; and communication competence, *p* = 0.007).

### Student engagement, attitudes, satisfaction, or perceptions toward TBL

Student engagement or attitudes toward TBL were reported in 2 studies (TBL = 89; LBL = 87) (21). Yang et al. (21) reported positive attitudes toward TBL and high levels of student engagement (TBL group 4.51 ± 0.54 vs. LBL group 4.28 ± 0.57, *p* = 0.039).

Student satisfaction or perceptions of TBL were reported in 4 studies (TBL = 190; LBL = 176) (11, 20, 40, 41). Xu et al. (40) reported high levels of satisfaction (TBL group 108.44 ± 9.97 vs. LBL group 103.72 ± 6.68, *p* < 0.01). Ulfa et al. (20) also revealed that TBL was associated with a higher level of nursing students' satisfaction (TBL group 34.19 ± 3.26 vs. LBL group 19.81 ± 1.94, *p* < 0.01). About the perception of TBL, Sakamoto et al. (11) (TBL = 14; LBL = 11) found positive results while this benefit (*p* < 0.02) disappeared 1 month later. Han et al. (41) (TBL = 62; LBL = 62) reported a majority of students had a positive perception of TBL (82.3–93.5%).

### Risk of bias

The risk of performance bias (blinding of participants and personnel) and detection bias (blinding of outcome assessment) were considered as the domain most frequently rated as a source of bias, with five at unclear risk and five at low risk. The risk of selection bias (allocation concealment) was unclear in 4 studies and low in 6 studies. The selection bias (random sequence generation) was unclear in 3 studies and low in 7 studies. The attrition bias was high in one study, and the other bias was low (Table 4). Therefore, the overall risk of bias was considered moderate in the performance bias and detection domains and low in the other four domains.

**Table 4:** Methodologic quality assessment of included studies (RCT).

**Study (year)**	**Random sequence generation (Selection bias)**	**Allocation concealment (Selection bias)**	**Blinding of participants and personnel (Performance bias)**	**Blinding of outcome assessment (Detection bias)**	**Incomplete outcome data (Attrition bias)**	**Other bias**
Yang et al. (21)	Unclear	Unclear	Unclear	Unclear	Low risk	Low risk
Ulfa et al. (10) A1	Low risk	Low risk	Low risk	Low risk	Low risk	Low risk
Ulfa et al. (20) A2	Low risk	Low risk	Low risk	Low risk	Low risk	Low risk
Sakamoto et al. (11)	Low risk	Low risk	Low risk	Low risk	High risk	Low risk
Lee et al. (13)	Low risk	Low risk	Low risk	Unclear	Low risk	Low risk
Yang et al. (39)	Low risk	Low risk	Low risk	Unclear	Low risk	Low risk
Kim et al. (14)	Low risk	Low risk	Unclear	Low risk	Low risk	Low risk
Badiyepeymaie Jahromi et al. (42)	Unclear	Unclear	Unclear	Unclear	Low risk	Low risk
Xu et al. (40)	Low risk	Unclear	Unclear	Unclear	Low risk	Low risk
Han et al. (41)	Unclear	Unclear	Unclear	Low risk	Low risk	Low risk

## Discussion

### Summary of the evidence

This systematic review confirmed the effectiveness of TBL in different settings. TBL could significantly improve students' academic knowledge, clinical performance, competency skills, satisfaction, perceptions, and attitudes. The first significant advantage of this review was that we only enrolled RCT design trials because previous reviews of studies of TBL report on the relative lack of randomized controlled studies (9, 15, 43).

TBL usually contains 4 phases: (i) teacher-guided pre-class preparatory learning; (ii) assessing mastery of core knowledge through the Individual Readiness Assurance Process' (iRAT) and Team Readiness Assurance Process (tRAT) test; (iii) application of newly acquired knowledge to significant authentic problems through application exercises and then students defend their decisions with evidence in a discussion led by the teacher; (iv) provide a peer evaluation of team members (6).

### Knowledge or clinical performance

All the reported studies were in favor of TBL with regard to academic scores. Our findings were like the previous reviews (15, 43). Possible reasons are that students in the TBL group prepared themselves with an out-of-class study by reading the iRAT material before the in-class sessions. The use of tests at the beginning of the in-class sessions also improved students' independent learning and acquisition of prior knowledge while students in the traditional classroom were passive learners and were not prepared individually to study early before attending the in-class sessions. And tRAT can stimulate students to attain a better understanding of the materials, especially poorly prepared students, as they can learn from their peers through sharing and discussion.

Moreover, Ulfa et al. (20) found that TBL could retain the nursing knowledge gained and had higher scores than the LBL group at long-term periods, and the results of Sakamoto et al. (11) also enhanced the conclusion. Possible reasons were that the tRAT in TBL could improve students' understanding of a clinical topic, as real conditions enhanced their memory and sharpened their understanding. The integration of learning strategies such as TBL and clinical practice could yield a comprehensive understanding, and such integration can help with knowledge retention.

### Competencies

Nursing students are required to gain the knowledge, techniques, and attitudes necessary to effectively solve problems that are presented in various situations. Therefore, core competencies were indeed needed, which included not only the perceptual capabilities that enable successful problem-solving in clinical situations but also widely applicable and complex capabilities such as healthy attitudes toward the self, others, and the organization as well as practical social skills (13).

Our study confirmed the effectiveness of TBL in promoting the core competencies of nursing education. The results were similar to previous studies (15, 43). Of note, the tools used for competency evaluation varied from trial to trial. In Yang et al. study (21), they used a self-designed nursing competence scale that reflected the ability of the community to understand or assess nursing skills. In Ulfa et al. (10) study, Clinical Reasoning Evaluation Simulation Tool (CREST) was applied, which comprehensively assesses the student's ability to solve cases and the student's analytical thinking in linking signs and symptoms to appropriate diagnoses and actions according to the scenario provided. Clinical reasoning is the ability to integrate knowledge and critical thinking. In the TBL process, the application exercise applies the topic concepts, stimulating students to use their knowledge and to think critically, therefore enhancing their clinical reasoning ability. In Lee et al. (13) study, they used five tools that measured the abilities of clinical competence, problem-solving, communication competence, critical thinking, and self-leadership. Self-directed learning ability was another kind of nursing core competency, which was presented as mean and standard deviation in the Yang et al. (39) study and rank scale in Badiyepeymaie Jahromi et al. study (42). The reason why TBL effectively developed the core competencies (including self-leadership, clinical competence skills, problem-solving ability, and critical thinking ability) was that TBL could assist nursing students in integrating and applying their knowledge previously learned in courses now studied in advance. In addition, TBL enhances communication competence *via* interactions among team members. Implementing TBL has obvious cost saving implications since facilitator requirements could be reduced by approximately half whereas provided equivalent clinical expertise at the same time (6). It should be noted, however, the difficulties instructors have when implemented TBL, which were pre-class preparation, academic expertise requirements, as well as in-class control (5, 43).

### Student engagement, attitudes, satisfaction

Our study found that the nursing students in TBL group had better performance in student engagement with class and had a most positive attitude and higher satisfaction levels with their experiences. The classroom engagement survey (CES) was used to assess student engagement in class in Ulfa et al. study (20). CES contained eight items, scored on a five-point Likert scale, with total scores ranging from 5 to 40. The reason for the enhanced engagement ability in the TBL group is that students were asked to have a discussion, in contrast to the traditional lectures wherein the students only learned passively. Therefore, the TBL activities showed how TBL could promote classroom engagement. In Yang et al. study (21), learning attitude was reported, and the results of collaborative learning and team efficacy favored TBL while learning attitude and individual self-efficacy were similar. Possible reasons were that TBL works mainly on the collaborative ability to improve. Though we found relatively high satisfaction regarding TBL implementation, the willingness of students was negative when promoting TBL since they have to do more pre-class practice, which increases their burdens (41). Nevertheless, instructors also found TBL hard to promote, as it requires instructors to develop IRAT/GRAT questions and teaching scenarios and imposes additional academic burdens on staff.

Therefore, for TBL to be more actively adopted in nursing education, instructors will require a suitable curriculum and sufficient time to prepare the management of TBL sessions. And students should be provided with sufficient information on the TBL processes in addition to learning content and sufficient time to conduct self-directed learning in advance using pre-class assignments or regular class sessions.

### Limitations

The limitations of this study were as follows: first, most of the included RCTs reported different outcomes or the same outcome with different measurement tools. Therefore, quantitative results were absent. Second, though standard TBL procedures were applied in class, the durations of TBL varied a lot, which may influence the results. Third, we include only RCT studies to gain high-quality and reliable results. However, RCT cannot fully measure the full array of learner responses.

## Conclusion

In general, this review suggested that the TBL was an effective pedagogy in improving academic performance and general ability in nursing students despite the education level regarding the current reports. However, most of the RCTs were of moderate quality. High-quality trials are in need, and standard outcomes should be applied. We recommend that future studies focused on TBL also include qualitative and observational components to ascertain a broader array of behavioral, cognitive, and motivational outcomes more deeply and perhaps to elucidate the mechanism (s) by which TBL effects student learning.

## Data availability statement

The original contributions presented in the study are included in the article/Supplementary material, further inquiries can be directed to the corresponding author.

## Author contributions

QZ and ZW designed the conception of the manuscript. XT and YZ drafted the original version of the manuscript and drew the figures and tables. XT, QZ, and ZW revised the final version of the manuscript. All authors contributed to the article and approved the submitted version.
