# Supplementary material for: Team-based learning vs. lecture-based learning in nursing: A systematic review of randomized controlled trials
Source: Front Public Health. 2023 Jan 4;10:1044014. doi: 10.3389/fpubh.2022.1044014 (PMC9846052; doi:10.3389/fpubh.2022.1044014)
Supplement: Supplementary file 1 [file Table_1.DOCX]

**Appendix 1. Specific search strategy in PubMed/Medline**

| **SEARCH STRINGS:** |
| --- |
| #1 team-based learning AND nursing  #2 team-based learning AND nursing AND (learning outcomes OR competences OR skills)  #3(("team-based learning") AND nursing education) AND learning outcomes |
| #1 team based learning OR team-based learning  #2 ((((("Education, Nursing"[Mesh]) OR "Students, Nursing"[Mesh]) OR "Nursing/education"[Mesh])) OR nursing education) OR nursing students  #3 (learner attitudes) OR (((((learning outcomes) OR competences) OR skills) OR ((("Academic Success"[Mesh]) OR "Academic Performance"[Mesh]) OR "Social Skills"[Mesh])) OR ((("Educational Status"[Mesh]) OR "Learning"[Mesh]) OR "Educational Measurement"[Mesh]))  #4 ((((((((("Education, Nursing"[Mesh]) OR "Students, Nursing"[Mesh]) OR "Nursing/education"[Mesh])) OR nursing education) OR nursing students)) AND (((((learning outcomes) OR competences) OR skills) OR ((("Academic Success"[Mesh]) OR "Academic Performance"[Mesh]) OR "Social Skills"[Mesh])) OR ((("Educational Status"[Mesh]) OR "Learning"[Mesh]) OR "Educational Measurement"[Mesh]))) AND ("team based learning" OR "team-based learning") |
